# Malunited extra-articular distal radius fractures: corrective osteotomies using volar locking plate

**DOI:** 10.1007/s10195-014-0307-x

**Published:** 2014-07-15

**Authors:** Luigi Tarallo, Raffaele Mugnai, Roberto Adani, Fabio Catani

**Affiliations:** 1 Department of Orthopaedic Surgery, University of Modena and Reggio Emilia, Via del Pozzo 71, 41124 Modena, Italy; 2 Department of Hand Surgery, University Hospital of Verona, Verona, Italy

**Keywords:** Malunion, Fractures, Radius, Osteotomy, Volar, Angulated, Locking plate, Bone graft, DASH, Mayo

## Abstract

**Background:**

Multiple techniques for corrective osteotomy have been developed in recent years with the same aims: to improve the radiographic parameters and improve motion, pain and grip strength. Volar fixed-angle plates have added a new concept to the treatment of distal radius fractures thanks to the low morbidity of the surgical approach and the strength of the final construct, allowing early mobilization and return to function.

**Materials and methods:**

Between 2005 and 2012, 20 patients with symptomatic dorsally malunited extra-articular fractures of the distal radius underwent corrective osteotomy using a volar locking plate without additional bone graft. At a mean follow-up of 50 months, all the patients were clinically and functionally evaluated.

**Results:**

All measurements of pain, final range of motion and grip strength significantly improved compared with preoperative measurements. The mean preoperative DASH score reduced from 54 points preoperatively to 25 postoperatively. Based on the modified Mayo wrist score, we obtained 14 excellent and six good results. Palmar tilt improved from an average of 23° to 11°. Radial inclination improved from an average of 29° to 22°, and ulnar variance decreased from an average of 3.6 mm to 0.9 mm. There were two cases of transient median neuroapraxia that resolved before the 6-week follow-up appointment. No other major complications, including non-union and infection, were observed.

**Conclusion:**

The volar approach and locking plate, without necessarily the use of bone grafting, proved to be an effective approach for addressing symptomatic and even severe deformities of the distal radius.

**Type of study/level of evidence:**

Therapeutic IV

## Introduction

Fractures of the distal radius are extremely common injuries and the outcome differs depending on the type of fracture. Normally stable distal radius fracture is treated non-operatively with a favorable result. In the other hand, unstable fracture easily becomes malunited with inadequate treatment. Malunion of the distal radius usually occurs following conservative treatment. The most common deformity following a malunited extra-articular fracture of the distal radius is the loss of the normal palmar tilt of the articular surface in the sagittal plane, and loss of length relative to the ulna. Once angulation of the distal articular surface of the radius becomes greater than 25–35° in the sagittal plane, Fernandez recommended corrective osteotomy [1, 2]. Several biomechanical studies have demonstrated abnormal wrist contact pressure with extra-articular deformity that can predispose to arthritis [3]. An increased contact pressure across the ulna is due to the loss of radial height, especially when the articular surface of the radius has a dorsal angulation. The anatomical position of the distal end of the radius with respect to the carpus and the ulna head is the key to obtaining normal wrist biomechanics. A wrist with a normal motion has about 120° of wrist flexion and extension, 50° of wrist radial and ulnar deviation, and 150° of forearm rotation. The radius carries 80 % of the axial load through the wrist, and the distal ulna only 20 % [4]. Malalignment of the distal radius due to an osseous deformity affects the normal load transmission, causing a limitation in the extension-flexion arc of motion. Multiple techniques for corrective osteotomy have been developed in recent years with the same aims: to improve the radiographic parameters and improve motion, pain and grip strength. Authors such as Fernandez [2] have described the traditional treatment of osteotomy and dorsal plating with bone graft for dorsal angulated malunions. These techniques guarantee good restoration of the anatomy and relieve pain, but have sometimes been associated with irritation or rupture of extensor tendons. Volar fixed-angled plates have added a new approach to the treatment of distal radius fractures thanks to the low morbidity of the surgical approach and the strength of the final construct, allowing early mobilization and return to function [5]. The aim of this study is to report the outcomes of a cohort of patients affected by symptomatic dorsally malunited extra-articular fractures of the distal radius who underwent corrective osteotomy using a volar locking plate without additional bone graft.

## Materials and methods

**Fig. 1:**
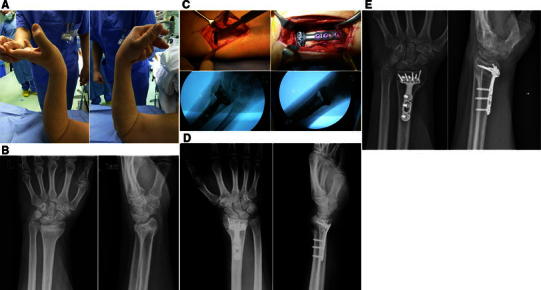
Case 1. **a** Preoperative range of movement evaluation. **b** Preoperative X-rays showing the dorsal inclination of articular surface of the distal radius.  **c** Intraoperative view showing the osteotomy of the radius, the synthesis and the final correction under fluoroscopy. **d** X-rays 30 days after surgery showing the dorsal gap with early signs of refilling. **e** Good healing of the osteotomy with complete refilling of the dorsal gap at 3 months after surgery

**Fig. 2:**
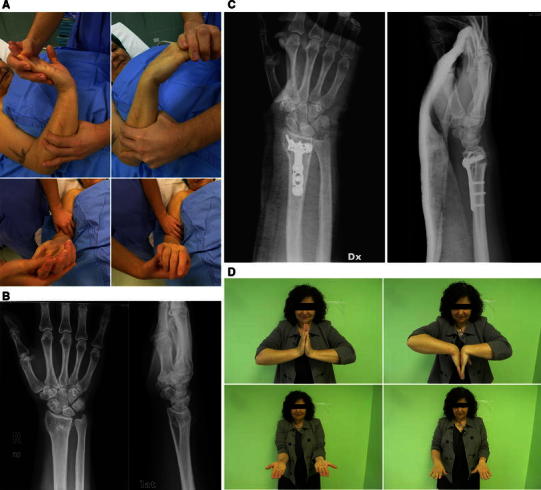
Case 2. **a** Range of motion evaluation before surgery with significant reduction of flexion. **b** Preoperative X-rays showing the dorsal inclination of articular surface of the distal radius. **c** Postoperative X-rays showing the correction of the dorsal deformity achieved. **d** Good functional recovery at 2 months after surgery

This prospective study was performed between 2005 and 2012. Inclusion criteria were a malunion following conservative treatment, with a dorsal tilt of the distal radial articular surface of more than 20°, articular displacement of more than 2 mm or incongruity of the distal radio-ulnar joint due to shortening of the distal radius in association with wrist pain and poor wrist range of movement. A total of 20 patients (8 women, 12 men) with a mean age of 40 years (range 17–64) were included in this study. The dominant arm was involved in 13 patients and the non-dominant arm in seven. Anesthesia was obtained by axillary nerve block. In all patients, a volar approach to the distal radius was performed. A longitudinal incision along the flexor radialis carpi was made. The radial artery was preserved and dislocated radially. The pronator quadratus was released using an “L” incision from the radial insertion. After exposure of the volar margin of the distal radius, the distal portion of the DVR^®^ plate (Hand Innovation) was held against the distal radius with K-wires. At this stage fluoroscopy was necessary to identify the correct positioning of the plate on the volar surface of the radius and for planning the level of the osteotomy. The plate was then removed after marking the position of the plate and the line of the osteotomy. Once the plate was removed and the osteotomy was performed, the fragments were distracted using a small osteotome as a lever to correct the deformity in lateral view under fluoroscopy. The plate was then fixed to the radius on the distal fragment, allowing the correct volar tilt and radial inclination. Once the plate was fixed on the distal radius fragment, the oval hole of the plate was used to center the proximal axis of the plate and a screw was inserted to hold the plate to the radial shaft. In this way the preformed shape of the distal part of the plate helps to correct the dorsal tilt, and under fluoroscopy the surgeon can correct the radial inclination and radial lengthening. The remaining cortical screws were inserted to complete the implant (Figs. 1, 2). The pronator quadratus was sutured back into place, covering the plate. All the surgical procedures were performed by a single surgeon. All patients were placed in a palmar plaster splint for 15 days before starting rehabilitation.

It is our routine practice to evaluate all patients clinically at 2 weeks, and both clinically and radiologically at 1 and 3 months. Moreover, between June and October 2013 all the patients were clinically reviewed. The clinical assessment included the analysis of passive range of motion (ROM), grip strength, pain level during activities of daily living evaluated with a 10-cm visual analogue scale (VAS), and functional evaluation using disabilities of the arm, shoulder and hand (DASH) [6] and the Mayo elbow performance score (MEPS). The total MEPS score ranges from 5 to 100 points, with higher scores indicating better function. If the total score is between 90 and 100 points, function can be considered excellent; between 75 and 89 points, good; between 60 and 74 points, fair; less than 60 points, poor [7].

Grip strength was measured with a Jamar dynamometer (Asimov Engineering Corp., Santa Monica, CA, USA), and wrist ROM using a goniometer.

All the radiographic measurements, including palmar tilt, radial inclination and ulnar variance, were performed on the last follow-up X-rays using a picture archiving and communication system (PACS software, Fuji Synapse). Time of union was determined according to both radiological and clinical parameters. Radiological criteria included: bridging of the fracture site by bone, callus or trabeculae; bridging of the fracture seen at the cortices; and obliteration of the fracture line or cortical continuity. Clinical criteria were represented by the patient’s ability to bear weight on the injured limb and perform activities of daily living, and the presence of pain at the fracture site upon palpation and physical stress. Moreover, possible early or late complications such as non-union, infection, tendon rupture or tendon irritation, and nervous lesions were documented.

## Results

All osteotomy sites united at a median of 4 months (range 2–5 months) after surgery. The mean duration of follow-up was 50 months (range 20–75 months). All measurements of pain, final range of motion and grip strength significantly improved at the last follow-up compared with preoperative measurements (Table 1). The mean preoperative DASH score reduced from 54 points (range 27–70) preoperatively to 25 (range 12–36) postoperatively.

**Table 1:** Summary statistics

	Grip strength (kg) Mean (SD)	Range of motion				Pain	Functional outcome		Radiographic evaluation		
		Extension (°) Mean (SD)	Flexion (°) Mean (SD)	Supination (°) Mean (SD)	Pronation (°) Mean (SD)	VAS Mean (SD)	DASH Mean (SD)	Mayo Mean (SD)	Palmar tilt (mm) Mean (SD)	Radial inclination (mm) Mean (SD)	Ulnar variance (mm) Mean (SD)
Preoperative	10.9 (2.9)	39.3 (11.2)	44.8 (16.8)	16.0 (7.5)	75.3 (14.5)	1.1 (1.3)	53.8 (13.5)	46.9 (10.4)	23.1 (6.5)	29.0 (6.9)	3.6 (0.3)
Postoperative	26.7 (10.5)	70.3 (9.2)	60.3 (17.8)	79.8 (15.5)	84.0 (7.2)	0.3 (0.6)	25.3 (7.7)	91.7 (5.0)	11.3 (4.4)	22.3 (6.1)	0.9 (0.6)
*P*	0.000^b^	0.000^b^	0.000^b^	0.000^b^	0.000^b^	0.028^a^	0.000^b^	0.000^b^	0.000^b^	0.000^b^	0.000^b^

^a^Wilcoxon test

^b^Paired samples *t* test

Based on the modified Mayo wrist score, we obtained 14 excellent and six good results. Palmar tilt improved from an average of 23° to 11°. Radial inclination improved from an average of 29° to 22°, and ulnar variance decreased from an average of 3.6 mm to 0.9 mm. There were no intraoperative complications noted. There were two cases of transient median neuroapraxia that resolved before the 6-week follow-up appointment. No other major complications, including non-union and infection, were observed.

## Discussion

Many studies have demonstrated that corrective osteotomy which restores anatomical configuration can effect an improvement in wrist function, forearm rotation, grip strength and pain [8]. Usually, an opening wedge osteotomy using dorsal plates and bone grafting has been performed for malunited Colles’ fractures [9]. However, when dorsal plates are used, a high incidence of plate removal has been reported because of painful hardware, tendon rupture and/or irritation [10–13]. There are several advantages to using a volar approach in the treatment of a malunited Colles’ fracture. If the dorsal compartments are not disturbed, the volar cortex can be fixed directly with a volar plate. Moreover, according to Malone et al. [14], the rigid characteristics of the volar locking plates are strong enough to avoid the requirement of structural bone grafting.

Donor site morbidity, especially at the iliac crest, has been well described and minor complications such as persistent pain at the harvest site, superficial sensory nerve injury, superficial hematoma or seroma and superficial infection have been reported [15]. Moreover, a volar approach is easier than a dorsal approach and the reduction of the volar cortex is simple because of less comminution and the advantage of direct vision [16]. The present study showed that a corrective osteotomy using a volar locking plate without the use of bone grafting could effectively produce a significant improvement in wrist function in patients treated for extra-articular distal radius malunion. We obtained an excellent correction of deformity based on radiographic parameters, with low morbidity and no non-unions, hardware failure or need for hardware removal. Our results are in line with those reported by Mahmoud et al. [17], who treated 30 malunited dorsally-angulated radii using fixed-angle volar locking plates without bone grafting, obtaining at a mean 18-month follow-up radiological evidence of union, correction of the deformity, and clinical and functional improvement in all cases. In particular, the improvement in the DASH and Mayo scores obtained in the present study was 28.5 and 42.8 points, respectively, compared with the 21.6 and 22.7 points reported by Mahmoud et al. [17]. These differences in functional outcome can probably be explained by the longer follow-up period of the present research. Favorable results have also been reported in numerous studies following volar locking plates with additional bone graft [18, 19]. The volar approach and the use of locking plates is an extremely effective and safe technique; in fact, the use of fixed-angle locking plates reduces the risk of postoperative bone displacement, and requires a shorter immobilization time [20, 21]. Moreover, the mechanical strength provided by this construct does not necessarily require the use of bone grafting. We therefore believe that the volar approach and locking plate, without necessarily the use of bone grafting, is an effective technique for addressing symptomatic and even severe deformities of the distal radius, and should be preferred especially in elderly patients with poor bone quality and with increased medical co-morbidities that may contraindicate the harvesting procedure, due to the longer operative time and the higher risks of bleeding and infection.

## References

[1] Fernandez DL (1988). Radial osteotomy and Bowers arthroplasty for malunited fractures of the distal end of the radius. J Bone Joint Surg Am.

[2] Fernandez DL (1993). Malunion of the distal radius: current approach to management. Instr Course Lect.

[3] Pogue DJ, Viegas SF, Patterson RM, Peterson PD, Jenkis DK, Sweo TD, Hokanson JA (1990). Effects of distal radius fracture malunion on wrist joint mechanics. J Hand Surg Am.

[4] Werner FW, Glisson RR, Murphy DJ, Palmer AK (1986). Force transmission through the distal radioulnar carpal joint: effect of ulnar lengthening and shortening. Handchir Mikrochir Plast Chir.

[5] Tarallo L, Mugnai R, Zambianchi F, Adani R, Catani F (2013). Volar plate fixation for the treatment of distal radius fractures: analysis of adverse events. J Orthop Trauma.

[6] Hudak PL, Amadio PC, Bombardier C (1996). Development of an upper extremity outcome measure: the DASH (disabilities of the arm, shoulder and hand) [corrected]. The Upper Extremity Collaborative Group (UECG). Am J Ind Med.

[7] Morrey BF, An KN, Chao EYS, Morrey BF (1993). Functional evaluation of the elbow. The Elbow and its Disorders.

[8] Jupiter JB, Ring D (1996). A comparison of early and late reconstruction of malunited fractures of the distal end of the radius. J Bone Joint Surg Am.

[9] Fernandez DL (1982). Correction of post-traumatic wrist deformity in adults by osteotomy, bone grafting, and internal fixation. J Bone Joint Surg Am.

[10] Schnur DP, Chang B (2000). Extensor tendon rupture after internal fixation of a distal radius fracture using a dorsally placed AO/ASIF titanium pi plate. Arbeitsgemeinschaft für Osteosynthesefragen/Association for the Study of Internal Fixation. Ann Plast Surg.

[11] Simic PM, Robison J, Gardner MJ, Gelberman RH, Weiland AJ, Boyer MI (2006). Treatment of distal radius fractures with a low-profile dorsal plating system: an outcomes assessment. J Hand Surg Am.

[12] Kamath AF, Zurakowski D, Day CS (2006). Low-profile dorsal plating for dorsally angulated distal radius fractures: an outcomes study. J Hand Surg Am.

[13] Jupiter JB, Fernandez DL (2002). Complications following distal radial fractures. Instr Course Lect.

[14] Malone KJ, Magnell TD, Freeman DC, Boyer MI, Placzek JD (2006). Surgical correction of dorsally angulated distal radius malunions with fixed angle volar plating: a case series. J Hand Surg Am.

[15] Arrington ED, Smith WJ, Chambers HG, Bucknell AL, Davino NA (1996). Complications of iliac crest bone graft harvesting. Clin Orthop Relat Res.

[16] Yasuda M, Masada K, Iwakiri K, Takeuchi E (2004). Early corrective osteotomy for a malunited Colles’ fracture using volar approach and calcium phosphate bone cement: a case report. J Hand Surg Am.

[17] Mahmoud M, El Shafie S, Kamal M (2012). Correction of dorsally-malunited extra-articular distal radial fractures using volar locked plates without bone grafting. J Bone Joint Surg Br.

[18] Peterson B, Gajendran V, Szabo RM (2008). Corrective osteotomy for deformity of the distal radius using a volar locking plate. Hand (N Y).

[19] Sato K, Nakamura T, Iwamoto T, Toyama Y, Ikegami H, Takayama S (2009). Corrective osteotomy for volarly malunited distal radius fracture. J Hand Surg Am.

[20] Orbay J (2005). Volar plate fixation of distal radius fractures. Hand Clin.

[21] Orbay JL, Badia A, Indriago IR, Infante A, Khouri RK, Gonzalez E, Fernandez DL (2001). The extended flexor carpi radialis approach: a new perspective for the distal radius fracture. Tech Hand Up Extrem Surg.

